# SARS-CoV-2 Serological Investigation of White-Tailed Deer in Northeastern Ohio

**DOI:** 10.3390/v15071603

**Published:** 2023-07-22

**Authors:** Patricia A. Boley, Patricia M. Dennis, Julia N. Faraone, Jiayu Xu, Mingde Liu, Xiaoyu Niu, Stormy Gibson, Vanessa Hale, Qiuhong Wang, Shan-Lu Liu, Linda J. Saif, Scott P. Kenney

**Affiliations:** 1 Center for Food Animal Health, The Ohio State University College of Food, Agriculture and Environmental Sciences, Wooster, OH 44691, USA; 2 Veterinary Preventative Medicine, The Ohio State University College of Veterinary Medicine, Columbus, OH 43210, USAfaraone.7@osu.edu (J.N.F.); liu.6244@osu.edu (S.-L.L.); 3 Cleveland Metroparks Zoo, Cleveland, OH 44109, USA; 4 Ohio Department of Natural Resources Division of Wildlife, Columbus, OH 43299, USA

**Keywords:** SARS-CoV-2, COVID-19, deer, seroprevalence, ELISA, Ohio

## Abstract

Coronaviruses are known to cross species barriers, and spill over among animals, from animals to humans, and vice versa. SARS-CoV-2 emerged in humans in late 2019. It is now known to infect numerous animal species, including companion animals and captive wildlife species. Experimental infections in other animals have established that many species are susceptible to infection, with new ones still being identified. We have developed an enzyme-linked immunosorbent assay (ELISA) for detecting antibodies to SARS-CoV-2 nucleocapsid (N) and spike (S) proteins, that is both sensitive and specific. It can detect S antibodies in sera at dilutions greater than 1:10,000, and does not cross-react with antibodies to the other coronaviruses tested. We used the S antibody ELISA to test serum samples collected from 472 deer from ten sites in northeastern Ohio between November 2020 and March 2021, when the SARS-CoV-2 pandemic was first peaking in humans in Ohio, USA. Antibodies to SARS-CoV-2 were found in serum samples from every site, with an overall positivity rate of 17.2%; we further compared the viral neutralizing antibody titers to our ELISA results. These findings demonstrate the need to establish surveillance programs to monitor deer and other susceptible wildlife species globally.

## 1. Introduction

The announcement that the United States has exceeded one million deaths due to SARS-CoV-2 [1] is a grim reminder that the pandemic prevails, despite recent reports of lower hospitalization/death rates. Although several vaccines have been in use for over a year, the disease has caused over 6.9 million deaths worldwide [2]. 

Coronaviruses have demonstrated a propensity for crossing species barriers, including animal-to-animal spread, animal-to-human spread, and human-to-animal spread. In 2002, a related zoonotic coronavirus, severe acute respiratory syndrome (SARS)-CoV jumped from bats, to potentially civet cats, to humans, causing 8098 confirmed cases, with 774 deaths, in 37 countries [3]. The Middle East respiratory syndrome coronavirus (MERS-CoV) emerged more recently, jumping repeatedly from dromedary camels to humans, causing 2604 respiratory cases, with 936 deaths to date [4]. Since the emergence of SARS-CoV-2 in late 2019, more than 15 susceptible [5] host species have been identified, and new ones are still being identified [6]. The transmission of SARS-CoV-2 to companion/pet animals (dogs, cats, hamsters, and ferrets) has been reported [7]. Cases in captive wildlife species, including big cats (lions, tigers, snow leopards, etc.), gorillas, otters, and minks have also been identified [7]. Experimental infections have established that other species are susceptible to SARS-CoV-2 infection including, but not limited to, hamsters, skunks, raccoons, rabbits, white-tailed deer, and non-human primates [8,9,10,11,12].

**Figure 1:**
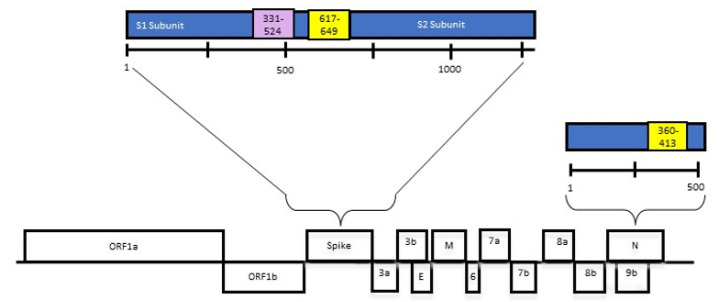
The organization of the SARS-CoV-2 genome by protein structures. The units of measure are amino acids. The yellow rectangles depict the fragments of spike and nucleocapsid (N) used in the ELISA. The purple rectangle depicts the receptor binding domain of the spike protein. This schematic representation is not indicative of the physical size of proteins.

Approximately two-thirds of the 5′ end of a coronavirus genome encodes non-structural proteins, whereas the 3′ end of the genome encodes structural and accessory proteins. The structural proteins consist of spike (S), envelope (E), membrane (M), and nucleocapsid (N) proteins (Figure 1) [13]. Of these structural proteins, two are substantially immunogenic, the N and S proteins [14]. Serological assays to detect binding and virus neutralizing antibodies have been developed against distinct domains of both the N protein and S protein, including the S1 subunit [15,16,17].

In this paper, we describe our development of enzyme-linked immunosorbent assays (ELISAs) for the detection of antibodies to the N and S protein domains of SARS-CoV-2. We validated the assays’ specificity against a large cohort of serum samples from other human and animal coronaviruses. We then field-tested the ELISA on a large cohort of samples from white-tailed deer from northeastern Ohio, and compared the ELISA results with virus neutralization assays. Deer from this cohort exhibited positivity for SARS-CoV-2 RNA in nasal swabs using RT-PCR, with an overall rate of 35.8% [18]. White-tailed deer have been experimentally infected, and have demonstrated the ability to transmit the virus vertically [19] and to sentinel animals [11]. Free-ranging deer with antibodies for SARS-CoV-2 in other areas of the U.S. have also been reported [20,21]. Multiple spillover events corresponding to emerging variants in the human population have been introduced into the deer population [22]. Emerging research has also detected divergent strains emerging in the virus circulating among deer, with the potential for transmission back to humans [23]. In addition, new studies have shown that deer may serve as a reservoir for SARS-CoV-2 variants of concern thought to be nearly extinct in the human population, necessitating further study of this new reservoir host.

## 2. Materials and Methods

### 2.1. Recombinant Proteins

**Figure 2:**
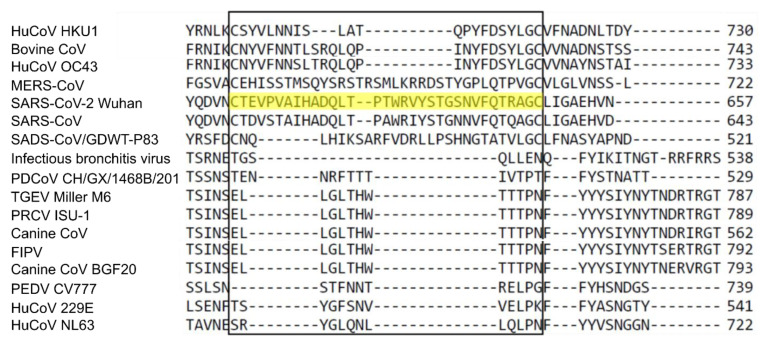
Amino acid sequence alignment comparison for the spike epitope (highlighted) between multiple coronaviruses. The sequences utilized in the alignment are HKU1 (ABD75553.1), bovine CoV (AAA66399.1), HuCovOC43 (ABU39940.1), MERS-CoV (AKQ21083.1), SARS-CoV-2 Wuhan (QRR29640.1), SARS-CoV (AAR07626.1), swine acute diarrhea syndrome CoV (QID98976.1), infectious bronchitis virus (AAW33786.1), porcine deltacoronavirus (QHI08611.1), transmissible gastroenteritis virus (TGEV Miller M6)(ABG89301.1), porcine respiratory CoV (PRCV-ISU1) (ABG89317.1), canine CoV (QJI07171.1), feline infectious peritonitis virus (FIPV) YP_004070194.1, canine coronavirus BGF20 (ADU17734.1), porcine epidemic diarrhea virus (PEDV CV777)(NP_598310.01), HuCoV229E (AAK32190.1), and HuCoVNL63 (APF29071.1).

**Figure 3:**
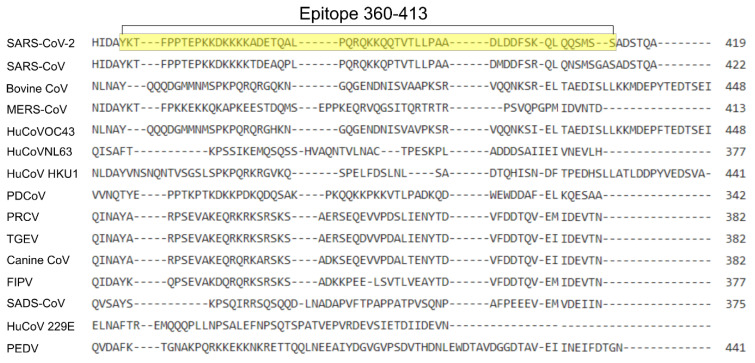
Amino acid sequence alignment comparison for the nucleocapsid epitope (highlighted) between multiple coronaviruses. Clustal omega was utilized to align the nucleocapsid genes from 14 different coronaviruses with SARS-CoV-2 N. The sequences utilized in the alignment are SARS-CoV-2 Wuhan (YP_009724397.2), SARS-CoV Urbani (AYV99827.1), bovine CoV(AAA42758.1), MERS-CoV (QYU59329.1), HuCovOC43 (QXL74890.1), HuCoVNL63 (ABK63972.1), human coronavirus HKU1 (ABG77571.1), porcine deltacoronavirus PDCoV (ANJ61325.1), PRCV-ISU1 (ABG89315.1), TGEV (APA05106.1), canine coronavirus (BAW32708.1), feline infectious peritonitis virus (FIPV) (BAC01159.1), swine acute diarrhea syndrome CoV (UAL80455.1), human coronavirus 229E (QNT54801.1), and porcine epidemic diarrhea virus (PEDV) (QQK84872.1).

Two nucleotide sequences, encoding for the amino acids 617–649 of the S1 subunit of the S protein (Figure 2) and amino acids 360–413 of the N protein of SARS-CoV-2 isolate USA-WA1/2020 (Genbank: MN908947.3) (Figure 3), were codon-optimized for bacterial expression, and synthesized commercially (Integrated DNA Technologies, IDT, Coralville, IA USA). The individual sequences were inserted into the bacterial T7 expression vector pRSETa (Invitrogen, Waltham, MA, USA), to produce recombinant peptides with the S or N epitope attached to a 6 X histidine tag and Xpress epitope tag. Restriction enzyme digestion and Sanger sequencing were used to verify the insertion. Recombinant proteins were produced using BL21 (DE3) chemically competent cells, via autoinduction [24]. The soluble fraction proteins were analyzed via SDS-PAGE and Western blotting. The bacteria were lysed with B-Per™ reagent (Thermofisher, Waltham, MA, USA) (5 mL/g), with 1 mM ethylenediaminetetraacetic acid (EDTA), and 1 mg/mL lysozyme. Briefly, the mixture of bacteria and reagents was pipetted up and down to homogenize, and incubated at room temperature (RT) with gentle rocking for 15 min. The tubes were centrifuged at 10,000× *g* for 10 min at RT. The supernatant was removed, and the insoluble portion was stored at −80 °C. For inclusion body purification, IB wash buffer (20.0 mM Tris-HCl, pH 7.5, 10.0 mM EDTA, 1.0% Triton X-100) was used to wash the insoluble material three times. IB solubilization buffer (50.0 mM CAPS, pH 11.0, 0.5% N-lauroylsarcosine, 0.1 M dithiothreitol) was incubated with the remaining insoluble material for 30 min at RT, on an end-over-end mixer. The remaining insoluble material was removed via centrifugation at 8000× *g* for 10 min. The soluble material was dialyzed against 20 mM Tris-HCl (pH 8.5) buffer using a slide-a-lyzer dialysis cassette (Thermo Fisher, Waltham, MA, USA). Then, a HisPur^®^ Ni-NTA purification kit (Thermofisher, Waltham, MA, USA) was used, according to directions. The final wash buffers were retained and tested using a SpectraMax Quickdrop Microvolume Spectrophotometer (Molecular Devices, San Jose, CA, USA) at A280 nm. The samples were run on SDS-PAGE, followed by Western blotting (Figure S1). This was followed by dialysis using a Slide-a-lyzer dialysis kit (Thermofisher, Waltham, MA, USA), as directed. The protein concentration was assessed using a Bio-Rad (Bio-rad, Hercules, CA, USA) protein assay kit, via the Bradford method. The protein lysates were subsequently aliquoted into smaller amounts, and stored at −80 °C.

### 2.2. Sodium Dodecyl Sulfate–Polyacrylamide Gel Electrophoresis (SDS-PAGE)

Recombinant proteins were analyzed with standard precast SDS-PAGE gels to assess protein purity and integrity. 1 µL of protein was mixed with Laemmli buffer containing β-mercaptoethanol, to obtain a final concentration of 2X. Samples were heated at 100 °C for 10 min then loaded into polyacrylamide gel 4–20% (Bio-rad, Hercules, CA, USA). Gels were stained with coomassie blue (Bio-rad, Hercules, CA, USA).

### 2.3. Western Blotting

To confirm specific protein expression in the soluble and insoluble fractions, a Western blot analysis was performed. Firstly, 25 µL of each sample was placed in a microcentrifuge tube with 6 µL 6X Laemmli buffer. The tubes were placed on a heat block at 100 °C for 10 min. The samples were then loaded onto a pre-cast 4–20% mini protein gel (Bio-rad, Hercules, CA, USA). The gel was run at 200V for 45 min, transferred to a PVDF membrane using a TransBlot^®^ Turbo™ system (Bio-rad, Hercules, CA, USA). The membrane was incubated in blocking buffer (Intercept, Li-Cor, Lincoln, NE, USA) in PBS, 1:1) with rocking for 2 h. The membrane was washed three times with phosphate buffered saline with 0.05% Tween 20 (PBST), and incubated with anti-Xpress antibody (Thermofisher, Waltham, MA, USA) at 1:1000 in blocking buffer at 4 °C overnight. After incubation, the membrane was washed three times with PBST, and incubated with horseradish-peroxidase (HRP)-conjugated goat anti-mouse-IgG at 1:1000 in blocking buffer, with rocking for 1 h at room temperature. The membrane was washed three times with PBST, and incubated with the CN/DAB substrate (Thermofisher, Waltham, MA, USA) to develop a colorimetric signal. The color development occurred after 30 min, and was quenched with water, and imaged.

### 2.4. ELISA

The enzyme-linked immunosorbent assays (ELISAs) were modified and optimized [25] to detect SARS-CoV-2 N- or S-specific IgG antibodies in serum. Two µg/mL of purified N, S, or empty plasmid lysate diluted in carbonate buffer (20 mM Na_2_CO_3_, 20 mM NaHCO_3_, pH 9.6) was bound to Nunc Maxisorp 96 well plates (Thermofisher, Waltham, MA, USA) at 50 µL per well at 4 °C overnight. The following morning, 150 µL of blocking buffer [5% nonfat dried milk (NFDM) in PBS with 0.1% Tween (PBST 0.1%)] was added to the antigen-coated wells, and incubated for 2 h at 37 °C. The plates were washed, and 50 µL serum, twofold serially diluted in blocking buffer, was added (1:100–1:3000 range for the CoV cross-reactivity studies; 1:10 to 1:10,240 for the deer samples). The plates were incubated for 1 h at 37 °C. After washing, 50 µL of HRP-conjugated secondary antibody [anti-swine, anti-bovine, anti-guineapig, anti-mouse, anti-chicken, anti-rabbit, or anti-deer (Seracare, Gaitherburg, MD, USA)] in 5% NFDM/PBST (0.1%) at a dilution of 1:400–1:3000 were added, and incubated at 37 °C for 1 h. The wells were washed with PBST (0.1%) five times between each step. Next, 3,3’,5,5’-tetramethylbenzidine (TMB) substrate (Seracare, Gaithersburg, MD, USA) was added, and incubated for approximately 10 min, and the reaction was stopped by adding 50 µL of 0.3 mol/L sulfuric acid. The plates were read at an absorbance of 450 nm, using a SpectraMax plate reader (Molecular Devices, San Jose, CA, USA). The statistical analysis was conducted using Graphpad Prism software (Graphpad, Boston, MA, USA). All experiments were conducted under the same conditions, with each sample tested in triplicate, and assays repeated three times.

For the testing of the field samples, the deer samples were heat inactivated for 30 min at 56 °C, and tested using a titration ELISA, starting with a 1:10 dilution, and serial twofold dilutions up to 1:10,240. A cutoff was established for each dilution, using 30 pre-COVID-19 deer serum samples from 2018, and using the method described by Frey et al. [26]

### 2.5. Reference Viruses, Sera, Anti-Sera, and Antibodies

The panel of human and animal CoVs used to test for cross reactivity is summarized in Table 1. They consisted of hyperimmune sera, normal sera, convalescent sera, and antibodies. Some were obtained commercially, and others were provided by our coauthors/collaborators as noted [25].

### 2.6. 50%. Plaque-Reduction Neutralization Test (PRNT_50_)

The detection of virus-neutralizing (VN) antibodies was performed as described previously, with modifications [27]. Briefly, 5 × 10^5^ Vero E6 cells (ATCC No. CRL-1586) per well in Dulbecco’s Modified Eagle’s Medium (DMEM) (Thermofisher, Waltham, MA, USA), supplemented with 1% penicillin–streptomycin (Thermofisher, Waltham, MA, USA) and 10% heat-inactivated fetal bovine serum (FBS) (Cytiva, Marlborough, MA, USA), were seeded in 24-well plates, and cultured in a cell culture incubator (37 °C with 5% CO_2_) until confluency was attained. To prepare the serum–virus mixture, 4-fold serially diluted serum samples were mixed with an equal volume of the SARS-CoV-2 strain USA-WA1/2020 (BEI Resources, Cat. # NR-52281), and the serum–virus mixtures were incubated at 37 °C for 1 h. A virus control (50 PFU of virus/well for the final inoculation), medium control, and positive and negative serum controls were included. The cell monolayers were washed twice with medium, and inoculated with serum–virus mixtures or controls. Each sample was tested in duplicate. The plates were incubated at 37 °C for 1 h. The inoculum was removed, and the cell monolayers were washed twice. Then, the cell monolayers were covered with 1% methylcellulose in MEM supplemented with 2% FBS, 1% nonessential amino acids (Thermofisher, Waltham, MA, USA), and 1% penicillin–streptomycin. At 4 dpi, the methylcellulose was removed, and the plates were fixed with 10% formalin, and stained with 0.2% crystal violet. After washing once with water, pictures were taken, and the plaques were counted. The VN titer of a serum sample was defined as the reciprocal of the highest dilution that resulted in at least a 50% reduction in the plaques based on the virus control wells.

### 2.7. Virus Neutralization Test (VNT)

To corroborate the ELISA results, a pseudotyped lentiviral vector assay was performed, using a D614G-containing SARS-CoV-2 spike. Virus neutralization assays were performed on 100 selected samples. Firstly, pseudotyped lentiviral vectors were produced in 293T cells by cotransfecting the pNL4-3-inGluc vector and D614G spike plasmid (GenScript Biotech, Piscataway, NJ, USA) in a 2:1 ratio, using a polyethyleneimine transfection (Transporter 5 Transfection Reagent, Polysciences, Warrington, PA, USA). The pseudotyped vectors were collected by harvesting the media off the producer cells at 48 and 72 h post transfection. The collected pseudotyped vector was then pooled and diluted 5-fold. Next, serum samples were serially diluted 4-fold (final dilutions 1:80, 1:320, 1:1280, 1:5120, 1:20,480, and the no-serum control). Then, 100 µL of the diluted pseudotyped vector was added onto the diluted sera, and incubated at 37 °C for 1 h. The vector/sera mixture was then added onto 293T-ACE2 cells. The supernatant was collected off the infected cells at 48 and 72 h post-infection, and used to measure the luciferase activity. Next, 20 µL *Gaussia* luciferase substrate (0.1 M Tris pH 7.4, 0.3 M sodium ascorbate, 10 µM coelenterazine) was added to an equal volume of infected cell medium, and the luminescence was immediately measured using a BioTek Cytation plate reader. The 50% neutralization titers (NT50) were determined using least-squares-fit, non-linear regression in GraphPad Prism 9 (San Diego, CA, USA). The data represent one replicate of the experiment for each sample.

### 2.8. Deer Sample Collection

Between November 2020 and March 2021, 472 free-ranging white-tailed deer originating from 10 study sites in northeast Ohio (USA) were euthanized as part of a deer population management program. The harvesting occurred at locations that were baited with whole-kernel corn for up to two weeks prior to each culling session, and additional deer were harvested opportunistically when they were observed away from the bait on a culling session day. Each day of the program, harvested deer carcasses were transported to a central processing point, where samples were collected. All samples were collected by one experienced veterinarian, who wore a facemask and gloves that were changed or washed between each sample. The blood was collected free-catch, following jugular vein puncture while the carcass was suspended head-down on a hanging scale. Blood was collected from each deer into 9 mL serum separator tubes. After collection, the samples were allowed to coagulate, and the serum was separated using centrifugation. The serum was immediately frozen at −20 °C, then transported on ice packs, and stored at −80 °C where it remained until testing was initiated. The sample collection was conducted post-mortem, meaning it was exempt from oversight by the Ohio State University Institutional Animal Care and Use Committee.

## 3. Results

### 3.1. Expression Constructs and Expression of Recombinant Proteins

We designed several SARS-CoV-2-specific constructs as synthetic double-stranded DNA (gblocks), which was synthesized commercially, based upon the specific portions of the S protein and N protein (Figure 1) described in previous reports [25] on ELISA cross-reactivity studies with SARS-CoV. Specifically, the N protein sequence (for amino acids 360–413) and spike protein sequence (for amino acids 617–649) were successfully cloned into the bacterial T7 expression vector pRSETa. After confirmation via sequencing, we produced the recombinant proteins, with the predicted sizes of 8.1 kDa (spike epitope) and 10.7 kDa (N epitope), using BL21 (DE3) chemically competent cells. We analyzed the proteins using SDS-PAGE and Western blotting, purified them, and reanalyzed them using SDS-PAGE (data not shown) and Western blotting (Figure S1). The multiple sequence alignment of the SARS-CoV-2 S protein with circulating animal and human coronaviruses, utilizing Clustal Omega software [28], showed that very little amino acid identity existed between the chosen epitopes for SARS-CoV-2 S, and those of the other coronaviruses, except for SARS-CoV, which showed 78.8% amino acid identity (Figure 2), while no other coronavirus S protein exhibited more than 25% identity to the chosen epitope. For the N epitope, SARS-CoV N shared 85.2% amino acid identity with SARS-CoV-2 N (Figure 3), while no other coronavirus N protein shared more than 18.5% identity with the chosen epitope.

### 3.2. ELISA Development and Optimization

We obtained commercial antibodies (Sinobiologicals) against SARS-CoV-2 N and SARS-CoV S1, to use as positive controls. The SARS-CoV S1 antibody was predicted to cross react with SARS-CoV-2 S, according to the manufacturer. Both commercial antibodies were produced in rabbits, so normal non-immunized rabbit serum was tested as a negative control. Checkerboard assays determined the optimal serum and secondary antibody dilutions, ranging from 1:100 to 1:3000. The commercial antibody against SARS-CoV-2 N was very sensitive, providing a strong signal, and was used at 1:3000. The commercial antibody against SARS-CoV S1 was less sensitive, and was used at a 1:100 dilution. The secondary antibody dilutions required optimization, and ranged from 1:500 to 1:2500, to provide consistent assay signals.

### 3.3. ELISA Testing for CoV S and N Protein Cross-Reactivity

**Figure 4:**
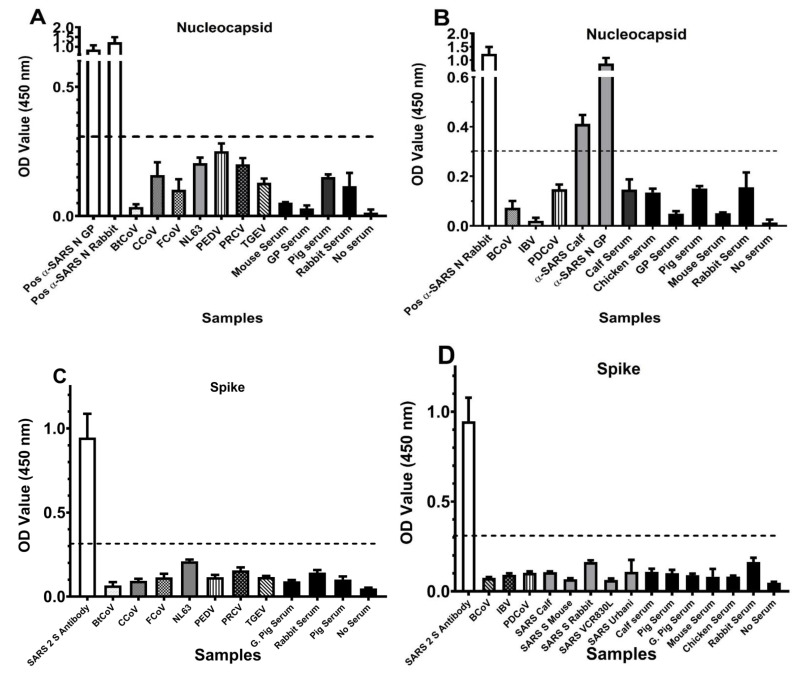
The graphed results of the ELISA cross-reactivity testing with other coronaviruses. (**A**) Nucleocapsid-based assay with alphacoronaviruses. (**B**) Nucleocapsid-based assay with beta-, delta-, and gammacoronaviruses, and control sera from several species. (**C**) Spike-based assay with alphacoronaviruses. (**D**) Spike-based assay with beta-, delta-, and gammacoronaviruses, and normal animal control sera. The broken line on each graph represents the negative cut-off.

We validated the specificity of the assays using a large cohort of reagents (Table 1). The only serum exhibiting any degree of cross-reactivity was the BEI serum number NR-10361 anti-SARS-CoV N, produced in guinea pigs and germ-free calf anti-SARS-CoV hyperimmune serum number NRC-2146, donated to the BEI repository by L.J. Saif. In both cases, cross-reactivity occurred only with the N-protein-based ELISA (Figure 4B, D). Because none of the available antisera tested were antigenically cross-reactive in the S fragment ELISA, we obtained a commercial antibody to the SARS-CoV-2 S protein to use as a positive control. Both the S and N ELISA assays showed either no cross-reactivity (S-ELISA) or minimal cross-reactivity (N-ELISA) when testing reference sera from multiple sources.

### 3.4. ELISA Results in Deer Samples

**Figure 5:**
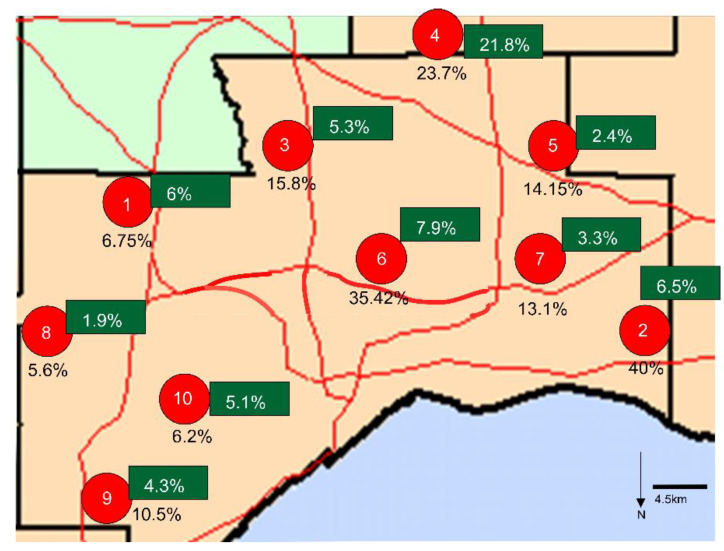
The ten study sites were spread across 1000 km^2^, with varying population density, in northeastern Ohio. The red dots represent the sampling locations, the unboxed percentages indicate the deer seropositivity rate in our study, and each green box indicates the reported percentage of the population reported as SARS-CoV-2-positive in each county in which the deer collection site was located, two weeks prior to the deer collection date.

**Figure 6:**
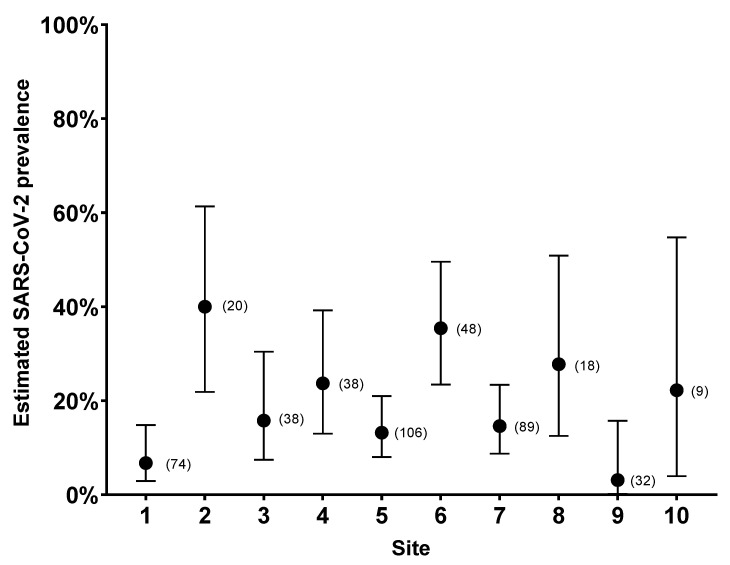
The prevalence of SARS-CoV-2 in white-tailed deer at each sampling site was estimated using a SARS-CoV-2 S peptide ELISA. The proportion of positive samples is shown with Wilson–Brown 95% confidence interval bars. The total number of samples per site is shown in parentheses.

We tested 472 deer serum samples collected between November 2020 and March 2021, from 10 sites in northeastern Ohio previously screened for viral RNA in nasal swabs by Hale et al. (Figure 5, [18]). A total of 81 samples tested positive via the spike-based ELISA, resulting in a seroprevalence of 17.2%, with a 95% upper confidence interval of 20.8%, and a 95% lower confidence interval of 14%, using the Wilson/Brown method. The results have been tabulated according to the sampling site (Figure 6). The ELISA antibody titers ranged from 1:10 to 1:10,240. Only one sample, from site 6, had a titer of 1:10,240. Site 6 had a high seroprevalence rate (*n* = 16/33, 48.9%) on the 28 January 2021 sampling date, and the samples that tested positive had high titers. At site 2, for the first sampling date in late January, three of four deer tested positive. At the second sampling at this site, in March, the positivity rate dropped to 31 percent (*n* = 5/16). The screening of the deer sera showed an overall seroprevalence of 17.2% from the test sites.

### 3.5. ELISA Epitope Sequence Stability

Despite the SARS-CoV-2 variants of concern averaging 12 spike amino acid substitutions between variants (alpha, beta, gamma, delta, and lambda), and 30 substitutions in omicron, the selected ELISA spike epitope genomic sequence remained relatively stable and relatively unaltered with the emerged variants of concern. When compared to the 291 full-length, high-coverage sequences available in GISAID on 13 July 2023, 282 retained the wild-type epitope sequence, five featured a serine to phenylalanine mutation at amino acid 640, and five strains featured single amino acid changes, including threonine 618 to isoleucine, valine 620 to isoleucine, proline 621 to serine, and serine 640 to cysteine. (Figure S2A). Further studies are needed to determine whether these mutations prevent antigen–antibody binding. Our initial nucleocapsid epitope may be more prone to amino acid substitutions over time, with only 155 of the 291 sequences retaining the original epitope sequence based upon the Wuhan genomic sequence (Figure S2B). Many of the alterations were associated with an aspartic acid to tyrosine change at amino acid 377 (D377Y), which occurred with the delta variant. Additional sequences with single amino acid changes or in conjunction with D377Y included lysine to asparagine at amino acid 373 in 5 of the 291 sequences; and lysine to arginine at amino acid 373, threonine to isoleucine at amino acid 379, serine to isoleucine at amino acid 413, alanine to valine at amino acid 414, serine to leucine at amino acid 416, and serine to leucine at amino acid 416, each found in a single deposited sequence (Figure S2B). From this, we suggest that the spike epitope is better- suited for current serological assays than N, as assays utilizing N may miss animals infected with delta variant viruses.

### 3.6. Neutralizing Antibody Titer Results in Deer Samples

The virus-neutralizing antibody titers were tested using a pseudovirus neutralizing assay (pVNT) and plaque reduction neutralization tests (PRNT_50_) on a subset of samples that were either positive in the ELISA, positive in RT-PCR, or belonged to a group of deer samples taken prior to COVID-19. The results, compared to those in the ELISA, are listed in Table 2. We found that our presumed false negative rate for the S-ELISA was approximately 5%, with one in eighteen samples having no ELISA positivity, yet it was able to neutralize SARS-CoV-2. Six samples out of eighteen (33%) were ELISA-positive but failed to produce neutralizing antibody titers.

## 4. Discussion

The antigenic cross-reactivity between CoVs has been examined previously between the original SARS-CoV and other animal and human CoVs [25,29]. The cross-reactivity between SARS-CoV and other CoVs has been attributed to the N protein [25,29,30]. Full-length SARS-CoV-N-protein-based serological assays, as well as commercially obtainable SARS-CoV-2 ELISA kits [31] have been reported to generate false positive results [32,33]. Commercially available and laboratory-developed ELISAs using the SARS-CoV N protein also show some degree of cross-reactivity with SARS-CoV-2, whereas the available ELISAs for SARS-CoV-2 testing utilizing the spike protein as the antigen have shown little false positivity, and no cross-reactivity [34,35,36]. Sequence homology data examining SARS-CoV-2 revealed that the N protein shares 94% and 90% homology with Bat-SL-CoV and SARS-CoV, while the S protein shares only 80% and 76%, respectively [37].

The delineation and definition of cross-reactivity previously identified localized regions of the SARS-CoV N protein that did not cross-react antigenically [25]. The region identified as non-reactive was identified as amino acid (aa) 360–412, which corresponded to aa 360–413 in the SARS-CoV-2 S protein. The analyses also determined that S, unlike N, was not antigenically cross-reactive.

We present a serological method for detecting SARS-CoV-2 infection and seroconversion in deer, based on previously reported successful methods to eliminate cross-reactivity with the original SARS-CoV and animal CoVs [25]. We tested for cross-reactivity with banked pre-pandemic deer sera, hyperimmune animal CoV, and SARS-CoV antisera, and commercial antibodies against various known coronaviruses affecting animals and humans. Our data showed negligible cross-reactivity with sera containing antibodies to other coronaviruses, with the exception of the anti-SARS-CoV nucleocapsid hyperimmune serum produced in guinea pigs and germ-free calves by the L.J. Saif lab, and donated to BEI. However, these sera did not cross-react with the SARS-CoV-2 spike fragment ELISA.

The 472 deer samples we tested were collected between November 2020 and March 2021. Potential confounding factors based on the use of convenience samples are the limited sample size, sampling dates, and geography. The sample size was not consistent between the sites and dates sampled, and ranged from 9 deer to 106 deer per site, and from 4 deer to 50 deer per date. The sample numbers, overall, were uneven (Table 3) making statistical comparisons difficult. The sampling dates also made it challenging to track the seroprevalence over time, as some sites were only sampled once, and some of the sites with numerous sampling dates took place on sequential days. Some of the sites are physically connected, making the contact and transmission of the virus between deer at adjacent sites possible.

Several studies have measured antibody titers in sera from experimentally infected deer [11,19], and samples collected from wild deer [20,21]. Palmer, et al. measured antibody titers in experimentally infected fawns up to 21 days post-infection. The spike- and nucleocapsid-based assays were similar, with the highest titers occurring at 21 days. The results were similar using a virus neutralization assay [11]. Cool et al. infected adult white-tailed deer, and found the antibody levels to be highest at seven days post contact (DPC) using an ELISA; however, the virus-neutralizing antibody levels were highest at 10 and 14 DPC, and similarly high at 18 DPC [19]. These differences could be attributed to differences in the immune response between adult deer and fawns. One sample, #1392, tested IgG-negative in the ELISA, but viral-RNA-positive in RT-PCR, and neutralizing-antibody-positive in both neutralization assays. We retested the sample using both S- and N-based versions of the ELISA, and both were negative. This could be indicative of the 5% false negative threshold for the assay congruent with existing commercial kits [38], although larger samples sizes must be evaluated in order to be accurate. An alternative explanation could be the timing of the infection relative to the blood-sampling, in which early antibody isotypes such as IgM might neutralize, but IgG has yet to be produced, and is thus not detected in our ELISA, which utilized anti-IgG secondary antibodies. We found that several deer samples testing positive in the ELISA were negative in both the assays for neutralizing antibodies. These findings have also been reported in the testing of human sera, and suggest that antibodies against SARS-CoV-2 S aa 360–413 could be generated faster than antibodies against other epitopes critical for neutralization. Supporting this observation, early studies demonstrated that most plasma samples recovered early in infection from convalescent humans did not contain neutralizing antibodies [39]. Nayak et al. examined antibodies to the whole SARS-CoV-2 virus at 20-, 40-, 60-, and 80-days post-PCR diagnosis in convalescent humans, and found that 78–90% had appreciable levels of IgG, IgM, and IgA antibodies, but only half of them had neutralizing antibody titers [40]. Additionally, studies identified nineteen antibodies that potently neutralized SARS-CoV-2 in vitro. Epitope mapping revealed that they were equally divided between those directed against the receptor-binding domain (RBD), and those directed against the N-terminal domain [41]. Another study noted that plasma IgG antibodies differed in their focus on RBD epitopes, recognition of alpha- and beta-coronaviruses, and contributions of avidity to increased binding and neutralization [42]. Studies conducted in wild deer populations can gauge seroprevalence at a given time, but it is currently not known for how long deer maintain circulating SARS-CoV-2 antibodies, or if these antibodies are associated with protection from re-infection. Finally, aligning the S protein from the genomic sequencing performed on viral RNA isolated from these deer, and other sequences deposited in Genbank from New York and Pennsylvania [11,18,43], with that of the SARS-CoV-2 isolate USA-WA1/2020 revealed several amino acid mutations within the RBD (Figure S3). These included glutamine to arginine at S amino acid 413 in all Pennsylvania deer samples, a glycine to valine substitution at position 445 in a single Pennsylvania isolate, leucine to arginine at position 454 in all Pennsylvania isolates, threonine to lysine at position 478 in all Pennsylvania deer sequences, and a single Ohio deer isolate with aspartic acid to glutamic acid at position 484. Interestingly, a 484 aspartic-acid-to-lysine (E494K) substitution has been associated with resistance in convalescent serum neutralization [44], which may also contribute to the discrepancies among the ELISA positivity, with a lack of neutralizing antibody titer in some samples.

Of the 360 deer in common between our ELISA testing and those nasal-swabbed and RT-PCR-tested by Hale et al. [18], 20 deer were positive in both assays, suggesting that these deer were either seroconverting in the final stages of viral clearance, or potentially represented a population incapable of clearing the virus efficiently, representing long-shedding individuals [45,46]. The seroprevalence of 17.2% for all samples was similar to that of the study recently conducted in Texas for neutralizing antibodies [20].

Deer are social animals living in herds, and could potentially spread the virus through nasal secretions, feces, and other social activities. Viable SARS-CoV-2 has been detected in human feces, and the RNA has been detected in wastewater [47]. The unchecked circulation of SARS-CoV-2 in deer populations represents a risk to humans if mutations in deer were to create a new variant that could escape immunity in humans. Populations of wild animals as reservoirs could also retain variants that are no longer currently circulating among humans, allowing them to reemerge in humans at a later date. Additionally, the closer genetic makeup of deer to other even-toed ungulates that are not currently highly susceptible to SARS-CoV-2, such as sheep, bison, pigs, and cows, may act as an intermediary that could foster more efficient replication in these agriculturally important species. Further monitoring of white-tailed deer populations, to determine the duration of antibodies, whether naturally infected animals can be reinfected, and the potential for viral evolution within these deer, is needed to understand the ramifications of SARS-CoV-2 circulation in deer herds. Interestingly, SARS-CoV-2 has not been detected in European deer populations, suggesting that anthropozoonotic transmission within the United States may be more prolific than abroad.

The high prevalence of exposure of white-tailed deer to SARS-CoV-2, the continued spillover from humans to deer, the potential for deer to serve as reservoirs for past variants of concern, and the potential for reintroduction to humans suggests that steps such as wearing N-95 masks should be taken by those who come into close contact with deer. People such as game hunters, deer farmers, and naturalists should use caution and avoid deer if SARS-CoV-2-positive, to break potential cycles of introduction.

## Figures and Tables

**Table 1:** Reagents tested for cross-reactivity.

Disease	Full Name	ID	Host	Source	Type	Assay
BCoV	Bovine coronavirus	B-12272 B574	Calf	LJS	HS	S and N
BCoV	Bovine coronavirus	Gp#99-10 Mebus	GP	LJS	HS	S and N
BtCoV	Bat coronavirus	HKU5.5N	Mouse	LJS	HS	S and N
CCoV	Canine coronavirus	2CoV UDC1	GP	LJS	HS	S and N
FCoV	Feline coronavirus	79-1146	GP	LJS	HS	S and N
HCoV	Human coronavirus	NL-63	GP	LJS	HS	S and N
IBV	Infectious bronchitis virus	M41	Chicken	LJS	HS	S and N
MERS	Middle East respiratory syndrome	PA5-81778	Rabbit	TF	Ab	N
PDCoV	Porcine deltacoronavirus	DC97	GP	LJS	HS	S and N
PDCoV	Porcine deltacoronavirus	DC163	Pig (Gn)	LJS	Serum	S and N
PDCoV	Porcine deltacoronavirus	DC173	Pig (Gn)	LJS	Serum	S and N
PEDV	Porcine epidemic diarrhea virus	PV1610	Pig (Gn)	QW	Serum	S and N
PEDV	Porcine epidemic diarrhea virus	PV1736	Pig (Gn)	QW	Serum	S and N
PRCV	Porcine respiratory coronavirus	ISU-1 PP12	Pig (Gn)	LJS	HS	S and N
SARS	Severe acute respiratory syndrome	VCR830L	Mouse	LJS	HS	S
SARS	Severe acute respiratory syndrome	Anti-S COV50	Mouse	LJS	HS	S
SARS	Severe acute respiratory syndrome	Urbani	Mouse	LJS	HS	S
SARS	Severe acute respiratory syndrome	NR-5469	Rabbit	BEI	HS	S
SARS	Severe acute respiratory syndrome	NR-10361	GP	BEI	HS	N
SARS	Severe acute respiratory syndrome	NRC-2146	Calf	LJS	HS	S and N
SARS	Severe acute respiratory syndrome	40150-T62	Rabbit	Sino	Ab	S
SARS 2	Severe acute respiratory syndrome 2	40588-T62	Rabbit	Sino	Ab	N
TGEV	Transmissible gastroenteritis virus	Purdue-ATCC	GP	LJS	HS	S and N
TGEV	Transmissible gastroenteritis virus	Purdue-ATCC	Pig (Gn)	LJS	HS	S and N
TGEV	Transmissible gastroenteritis virus	M2 H5	Pig (Gn)	LJS	HS	S and N
TGEV	Transmissible gastroenteritis virus	MM973	Pig (Gn)	LJS	HS	S and N

Abbreviations: GP = guinea pig, Pig (Gn) = gnotobiotic pig, LJS = Linda J. Saif, QW = Qiuhong Wang, HS = hyperimmune serum, Ab = antibody, S = spike protein, N = nucleocapsid protein, BEI = (BEI resources, Manassas, VA, USA), Sino = Sino Biological, Chesterbrook, PA, USA), TF = (Thermofisher, Waltham, MA, USA), accessed 1 July 2023.

**Table 2:** The SARS-CoV-2 S ELISA vs. viral neutralization results.

Sample #	Deer Tag #	ELISA Titer	PRNT50 Titer	pVNT	PCR+
1109	009	1:640	Neg	Neg	NT
1110	010	1:320	Neg	Neg	NT
1111	011	1:10	Neg	Neg	NT
1113	013	1:40	Neg	Neg	NT
1115	015	1:20	16	723.1	NT
1116	016	1:10	16	1021.9	NT
1118	018	1:10	64	1047.3	NT
1142	042	1:640	4	91.8	NT
1148	048	1:10	Neg	<80	NT
1150	050	1:320	16; 40 ^b^	285.3	NT
1162	062	1:640	4	252.9	NT
1288	188	Neg	Neg	Neg	Neg
1325	225	1:2560	Neg	<80	Neg
1392	292	Neg	64	661.8	Pos
1435	335	1:640	40	<80	Pos
1581	7 ^a^	Neg	Neg	Neg	NT
1601	148 ^a^	Neg	Neg	Neg	NT

^a^ Pre-COVID-19 samples, from 2018. ^b^ Sample tested twice. NT: not tested.

**Table 3:** The seroprevalence of SARS-CoV-2, by site and sampling date.

	Date of Collection	Samples	Positive	Estimated Seroprevalence	Upper 95% CI	Lower 95% CI
Site 1	2021-02-02	41	3	7.3%	19.43%	2.52%
	2021-02-25	33	2	6.1%	19.61%	1.07%
Site 2	2021-01-27	4	3	75%	98.71%	30.06%
	2021-03-03	16	5	31.3%	55.60%	14.17%
Site 3	2021-02-04	14	3	21.4%	47.59%	7.57%
	2021-03-02	24	3	12.5%	31.0%	4.34%
Site 4	2021-01-26	14	5	35.7%	61.24%	16.35%
	2021-02-17	16	2	12.5%	36.02%	2.22%
	2021-03-09	8	2	25%	59.07%	4.44%
Site 5	2021-01-19	11	3	27.3%	56.57%	9.75%
	2021-01-20	10	0	0%	27.75%	0%
	2021-01-21	32	6	18.8%	35.31%	8.89%
	2021-01-25	20	1	5%	23.61%	0.26%
	2021-02-09	8	2	25%	59.08%	4.44%
	2021-03-08	25	3	12%	29.96%	4.17%
Site 6	2021-01-28	33	16	48.5%	64.78%	32.50%
	2021-03-04	15	1	6.7%	29.82%	0.34%
Site 7	2021-02-22	20	3	15%	36.04%	5.24%
	2021-02-23	19	3	15.8%	37.57%	5.52%
	2021-02-24	50	7	14%	26.19%	6.95%
Site 8	2020-12-09	5	3	60%	92.89%	23.07%
	2021-02-16	13	2	15.4%	42.24%	2.73%
Site 9	2021-02-01	22	2	9.1%	27.82%	1.62%
	2021-03-01	10	0	0%	27.75%	0%
Site 10	2020-11-24	9	2	22.2%	54.74%	3.95%

The sample collection dates for all ten sites, indicating the number of serum samples collected from the white-tailed deer, and the number of those serum samples that screened positive in the S peptide ELISA assay. The estimated prevalence is shown with 95% confidence interval estimates (Wilson–Brown).

## Data Availability

Any additional data not published within the context of this article will be provided upon request by corresponding author.

## Supplementary Materials

The following supporting information can be downloaded at: https://www.mdpi.com/article/10.3390/v15071603/s1.
