# Supplementary material for: SARS-CoV-2 Serological Investigation of White-Tailed Deer in Northeastern Ohio
Source: Viruses. 2023 Jul 22;15(7):1603. doi: 10.3390/v15071603 (PMC10385782; doi:10.3390/v15071603)
Supplement: Supplementary file 1 [file viruses-15-01603-s001.zip › viruses-2497052-supplementary.pdf]

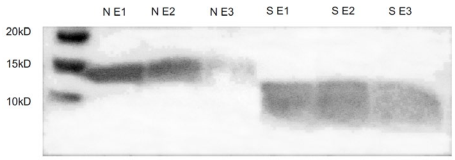

Figure S1 Western blot of spike and nucleocapsid peptides utilizing anti-Xpress antibody (N is nucleocapsid, S is spike, E is for elution fraction from nickel bead affinity column.

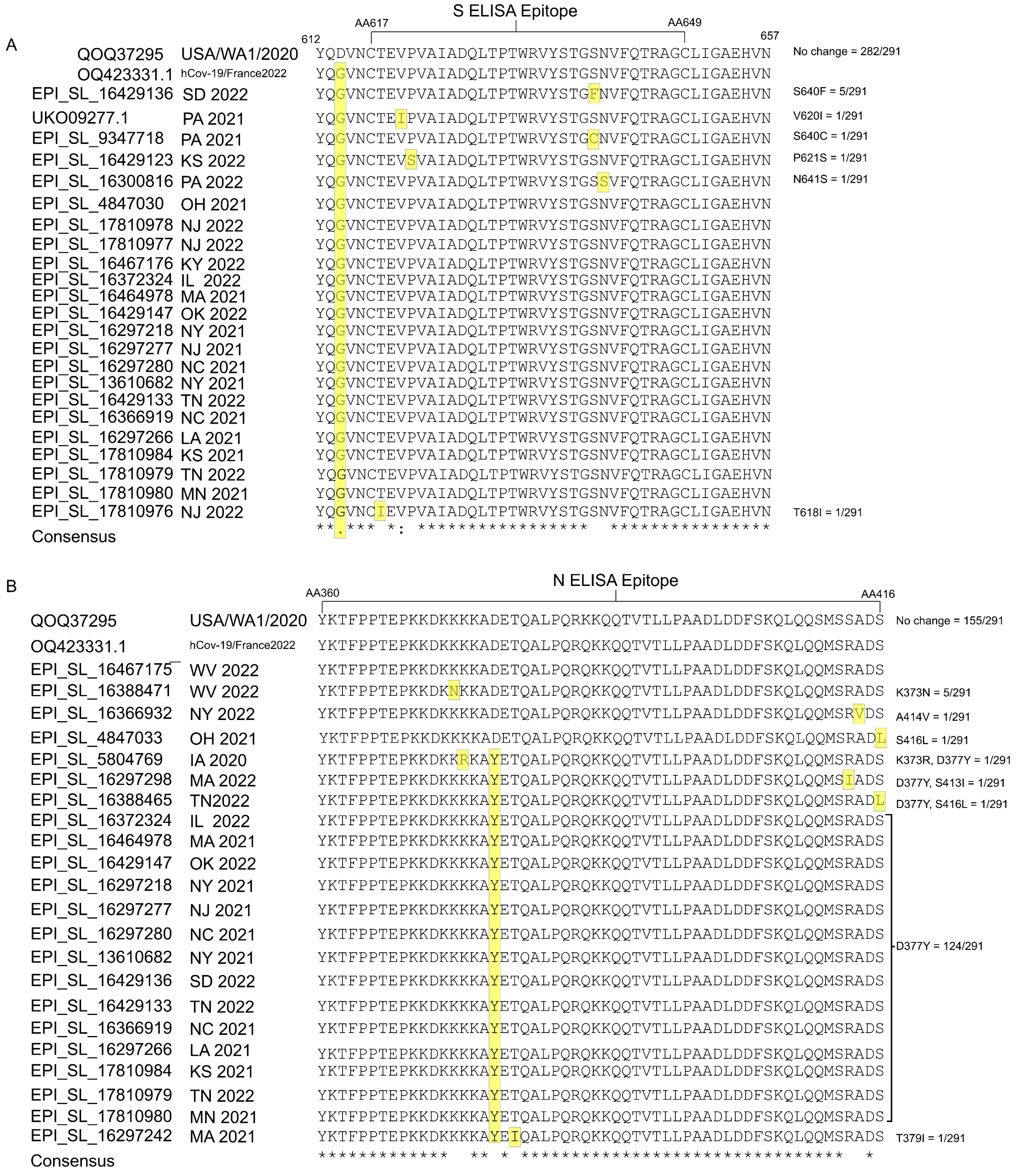

Figure S2 Sequence alignment of spike and nucleocapsid epitope sequences. (A) Spike ELISA epitope designed based upon the sequence of USA/Wa1/2020 aligned hCoV-19France 2022 and deer sample amino acids sequences generated from GISAID repository. (B) Nucleocapsid ELISA epitope designed based upon the sequence of USA/WA1/2020 aligned hCoV-19France 2022 and deer sample amino acids sequences generated from GISAID repository Columns from left to right are sequence identifier (either NCBI or GISAID) strain name or United States state from which sequence originated, year sequence was obtained, amino acid sequence, specific amino acid substitution identified, and prevalence among 291 full-length, high-coverage deer sequences deposited in GISAID. Asterisks denote conserved amino acid; colon denotes conserved amino acid change.

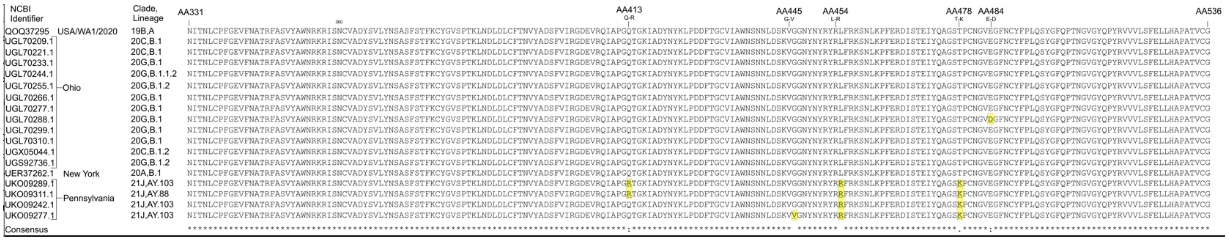

Figure S3 Clustal Omega alignment of S domain amino acids 331 through 536 of select deer isolates from 2020/2021. Amino acids changes from original USA/WA/2020 strain are depicted with yellow highlighting. Asterisks are identical sequences; colons are conservative amino acid changes, empty spaces are unconserved amino acid substitutions.
